# Experts’ perspectives on allergic reactions to emerging food sources, pollen and insects

**DOI:** 10.5414/ALX02611E

**Published:** 2026-03-17

**Authors:** Lea Faust, Sabine Dölle-Bierke, Veronika Höfer, Margitta Worm

**Affiliations:** 1 Division of Allergy and Immunology, Department Dermatology, Venereology and Allergy, Campus Charité Mitte, Universitätsmedizin Berlin, Berlin, Germany, and; 2 Division of Allergy, University Children’s Hospital and Children’s Research Center, University of Zurich (UZH), Zurich, Switzerland

**Keywords:** novel allergenic food, food allergens, legumes, edible insects, hemp seeds, jackfruit, cross-reactivity

## Abstract

The rising demand for sustainable diets has led to an increased consumption of alternative protein sources. While ecologically promising, these foods may pose new allergenic risks. We surveyed 127 European allergy experts regarding their clinical perception towards emerging allergenic food sources. Allergic reactions to non-priority allergenic foods were most frequently reported for legumes (83%), followed by hemp (33%), edible insects (21%), and jackfruit (20%). Experts highlighted risks related to cross-reactivity, particularly between edible insects and crustaceans or house dust mites, and between non-priority legumes and peanut, soy, or lupine. Legumes other than peanut, soy, and lupine, as well as edible insects and hempseeds/cannabis, were also rated as most clinically relevant for future practice. Experts also noted rising symptoms due to changes in pollen exposure and insect distribution linked to climate change. Our data underscore the need for the diagnosis of emerging allergenic sources.

## Introduction 

Global population growth and environmental concerns have intensified the demand for sustainable dietary protein sources [1]. In response, the concept of alternative proteins has gained increasing relevance, encompassing plant-based proteins, edible insects, and cultured meat [2, 3]. While some of these are classified as novel foods – defined as food not significantly consumed in the EU before May 1997 [4] – others involve the introduction of traditional ingredients in innovative dietary products (e.g., pea protein). In Western markets, the adoption of such products is largely driven by changing consumer expectations related to sustainability, health, and ethical values, leading to a diversification of protein sources and the growing market presence of alternative protein-based foods [5]. 

The introduction of alternative proteins raises important concerns [6], as they can act as primary sensitizers or trigger IgE-mediated cross-reactions in already sensitized individuals, particularly when sharing structural similarities with known allergens such as storage proteins, Bet v 1 homologues, or arthropod-derived proteins [7, 8, 9, 10]. 

In this study, we selected allergen sources that are either novel by regulatory definition or have recently gained relevance in Western dietary habits. For example, jackfruit (*Artocarpus heterophyllus*), offered as a meat alternative in vegan diets, may pose a risk of cross-reactivity in individuals with birch pollen or latex allergy [9, 11]. Similarly, hemp seeds, though not classified as novel food, have gained attention as a high-protein, nutritionally favorable ingredient, but their allergenic potential remains poorly characterized [12]. Several legumes that are generally underutilized, including pea, lentil, and fava bean, are already commonly used in plant-based protein alternatives and may pose a risk of cross-reactivity due to their botanical proximity to well-known allergenic foods such as peanut, soy, and lupine, or through de novo sensitization [7, 8]. Edible insects, such as *Acheta domesticus* (house cricket) and *Tenebrio molitor* (mealworm), are increasingly used in protein-rich products like flours, bars, and snacks. Their proteins share epitopes with tropomyosin and arginine kinase from crustaceans and mites, and concerns about clinically relevant cross-sensitization have been raised [10, 13, 14, 15]. 

To determine how frequently patients present with questions and/or allergic reactions related to these foods and to evaluate the experts’ opinions regarding their potential future relevance, we conducted a standardized online survey among experienced experts in allergy. 

## Materials and methods 

The expert survey (Supplemental Material) was conducted at the biannual International Conference of the Network of Online Registration for Anaphylaxis (NORA e.V.) in Berlin (2023 and 2025) and was additionally distributed to professionals working in the field of allergology. Only respondents with more than 1 year of clinical experience in allergology were included in the analysis. Data were collected from 127 experts (Table 1), most of whom were physicians (86%), followed by dieticians (13%) and one psychologist (0.8%). Among the physicians, the majority specialized in allergology (n = 55), either alone or in combination with other fields, or in dermatology (n = 19). The median clinical experience was 16 years (interquartile range (IQR) 5 – 28), and patients of all age groups were represented in the respondents’ clinical practice. Participants were based in several countries, with most practicing in Germany (38%), Austria (29%), Spain (6.3%), and Switzerland (5.6%). 72% of respondents were female. The primary patient groups included individuals with food allergies (88%), followed by those with pollen allergies (59%), insect venom allergies (46%), and drug allergies (44%). Data analysis was performed using R (version 4.3.1) [16], and the figures were created using R and GraphPad Prism (version 10.5.0). 

## Results 

### Allergic reactions, patient inquiries, and diagnostic testing across novel allergenic foods 

**Figure 1.**
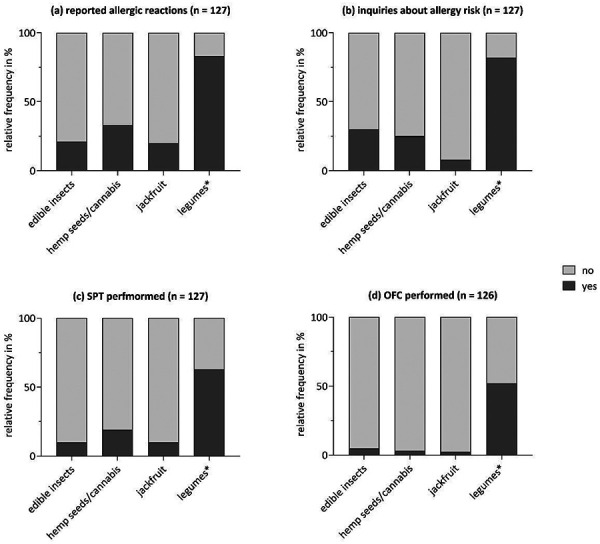
Relative frequency of (a) reported allergic reactions by patients, (b) patients inquiring about allergic risk, (c) performed skin prick tests (SPT), (d) performed oral food challenges (OFC) for each allergic food product (edible insects, hemp seeds/cannabis, jackfruit, legumes*). *Legumes other than peanut/soy/lupine.

27/127 experts (21%) stated that they had been consulted due to allergic reactions to edible insects, and 25 (20%) due to reactions to jackfruit. Hemp-related allergic reactions were seen by 42 experts (33%) and reactions to legumes other than peanut, soy, or lupine (hereafter referred to as legumes*) by 104 experts (83%) (Figure 1a); 18% of experts reported seeing more than 10 cases per year (Supplemental Figure E1). 

Patient inquiries about novel allergenic food sources most often concerned legumes* (78%, n = 103). Inquiries regarding edible insects (30%, n = 38), hemp seeds/cannabis (25%, n = 31), and jackfruit (8%, n = 10) were mentioned less frequently, but were still not uncommon (Figure 1b). 

Diagnostic testing with novel allergenic foods was uncommon (Figure 1c, d). For edible insects, 13 experts performed skin prick tests (SPTs), of whom 9 reported having positive reactions; 6 conducted oral food challenges (OFCs), with 4 reporting positive outcomes. For jackfruit, 12 experts performed SPTs (6 observed positive reactions), and 3 conducted OFCs (1 positive). For hemp seeds/cannabis, 25 experts performed SPTs (17 observed positives), and 4 conducted OFCs (all positive). Legumes* were tested more frequently: 80 experts performed SPTs (42 observed positives); 96 conducted specific IgE testing (84 positives; assessed only for legumes*), and 65 performed OFCs (61 positive). Detailed data are shown only for legumes* (Supplemental Figure E1). 

### Potential risk of cross-reactivities to novel allergenic food sources 

Experts were asked whether they actively advise patients with known allergies about potential cross-reactivities to novel allergenic foods. Counseling was most frequently reported for legumes*, followed by edible insects, hemp seeds/cannabis, and jackfruit (Table 2). 

### Future relevance of allergic reactions to novel allergenic foods 

**Figure 2.**
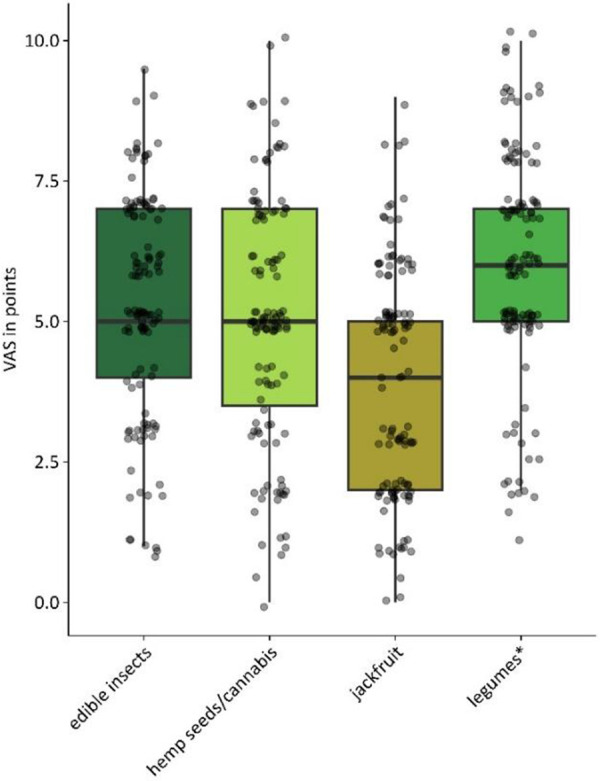
Expected increase in allergic reactions to novel allergenic food sources (edible insects, n = 125; hemp seeds/cannabis, n = 126; jackfruit, n = 125; legumes*, n = 126), presented as box plots (median and interquartile range) with individual values. Values are based on a visual analogue scale (VAS) from 0 to 10. *Legumes other than peanut/soy/lupine.

**Figure 3.**
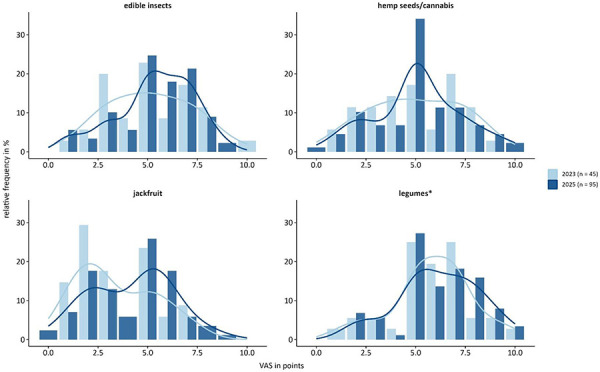
Expected increase in allergic reactions to novel allergenic food sources (edible insects, hemp seeds/cannabis, jackfruit, legumes*) based on visual analogue scale (VAS) from 0 – 10 in 2023 (n = 45) and 2025 (n = 95). Histograms show relative frequency (%) per group; overlaid lines represent smoothed density estimates. *Legumes other than peanut/soy/lupine.

Experts rated the future relevance of each allergenic food source on a visual analogue scale (VAS) from 0 to 10 (Figure 2). Edible insects and hemp seeds/cannabis both reached a median of 5 (IQR: 4 – 7 and 3.5 – 6.75). Between 2023 and 2025, ratings for edible insects shifted toward higher values, while hemp seeds/cannabis remained stable (Figure 3). Jackfruit scored lowest (median 4 (IQR: 2 – 5)), but increased in 2025. Legumes* achieved the highest scores (median of 6 (IQR: 5 – 7)) with small variation between the two assessment years. 

### Changes in pollen and venom allergen exposure 

Experts were asked about their recognition of changes in the duration of pollen and insect venom exposure. 34 experts reported that their patients had developed allergies to plant species not previously present in their country (Table 3). Among these, novel pollen allergens were most frequently reported for ragweed (53.5%, including mentions in combination with other pollens), followed by olive (29.5%) and tree of heaven (13.3%). 83 experts reported observing an extended period of symptoms in pollen-allergic patients due to a prolonged pollen season, most commonly for birch (23%), grasses (14%), a combination of hazel and alder (3.9%), ragweed (1.6%) and platanus (0.8%). 

21 experts reported cases linked to novel stinging insect species. The most frequently named were *Vespa velutina* and *Polistes* species. Less commonly mentioned were hornets, ants, various wasps, horseflies, mosquitoes, ticks, and ladybirds. Additionally, 30 experts reported an increased onset of allergic symptoms associated with prolonged insect season. 

## Discussion 

Our findings suggest an awareness of allergists regarding novel allergenic foods but also climate-induced changes in allergen exposure. The data show that exposure to emerging allergenic sources is not uncommon and indicate the need for standardized diagnostic tests. 

In the European Anaphylaxis Registry, non-priority legumes, particularly peas and lentils, were identified as relevant triggers of food-induced anaphylaxis [17]. In our study, these legumes received the highest future relevance scores and were the most frequently reported triggers, with 83% of experts having seen such cases in their practice. This aligns with increased consumption of plant-based products such as lentils, peas, and chickpeas, whose allergenic relevance varies depending on regional diets and habits [1, 18]. Although co-sensitization between legumes is common, clinically relevant cross-allergies remain rare [7, 19]. A cross-sectional study reported that among peanut- and soybean-allergic patients, clinically relevant co-allergies to other legumes were uncommon (≤ 16.7%), whereas co-sensitization, primarily due to IgE reactivity against 7S and 11S globulin fractions, was more frequent [7]. Patients allergic to green pea, lupine, lentil, or bean often had co-allergies to peanut (64.7 – 77.8%) or soybean (50 – 64.7%) [7]. Clinically relevant co-allergies could sometimes be explained by cross-reactivity, e.g., between green pea and lentil, whereas most peanut co-sensitizations were not clinically relevant [7]. In addition to cross-reactivity between legume storage proteins, in particular, peas pose a risk for birch pollen-allergic individuals. Ultra-processed pea ingredients, such as protein isolates, flour, and fibers, commonly used in plant-based drinks and protein shakes, can contain intact Bet v 1 homologous allergens, similar to Gly m 4 in soy, which may trigger anaphylaxis depending on the degree of processing [6]. Distinguishing serological sensitization from true clinical allergy is crucial in legume-allergic patients. In our study, 71% of specialists reported counseling on potential cross-reactivity; the questionnaire did not distinguish whether this referred to sensitization or clinical allergy. 

In Europe, cannabis may become an increasingly relevant allergen source as medical use expands and recreational use is gradually introduced in some countries. Can s 3, a heat- and digestion-stable non-specific lipid transfer protein (nsLTP), is the major allergen [20, 21, 22], with up to 95% of patients with confirmed cannabis allergy sensitized to purified Can s 3 [22]. In our study, 26% of experts routinely advised nsLTP-sensitized patients about potential allergic reactions to cannabis or hemp seeds. Risk perception of cannabis allergy was moderate, though legalization, increased cultivation, and expanded medical and nutritional use may influence future concern [12, 23, 24]. International data show that regions with legalized cannabis had the highest rates of allergy testing (71.4%) and patient consultations (82.9%) [25], suggesting that increased exposure and medical use may similarly influence awareness and consultation needs elsewhere. No commercial diagnostic tests are currently available for clinical use; the only industrial sIgE hemp assay is restricted to research purposes. Allergic reactions have been reported after inhalation, ingestion, or skin contact – during plant handling, processing, or via hemp-based foods, textiles, or cosmetics [12, 24, 26, 27]. Occupational exposure remains a concern for cannabis workers, who may develop respiratory symptoms, contact urticaria, dermatitis, or systemic reactions [28, 29]. 

Edible insects present a moderate risk for future allergic reactions according to our expert survey. Response patterns became more consistent over time, suggesting growing professional agreement on their potential clinical relevance. Although allergic reactions to edible insects remain rare in Western populations, Asian data indicate a higher prevalence: In Laos, 7.6% of insect consumers reported symptoms after consumption, and in China, 18% of fatal food-induced anaphylaxis, anaphylactic shock cases were attributed to edible insects [30, 31]. Despite limited public acceptance in Europe, earlier studies have shown that consumer willingness increases when insects are processed into unrecognizable forms like flour [32]. 30% of experts reported that they had seen patients who inquired about edible insect allergies. A major concern is the IgE cross-reactivity risk among patients with allergies to crustaceans and/or house dust mites. 43% percent of experts reported routinely advising such patients on potential cross-reactions. Molecular evidence suggests tropomyosin and arginine kinase as major pan-allergens that are highly conserved across arthropods [10, 33]. Cross-reactivity between shrimp and insect tropomyosin has been well demonstrated, particularly in shellfish-allergic patients [34]. Nevertheless, clinical evidence for cross-reactivity between house dust mite and insect allergens is lacking. Many insect proteins remain poorly characterized, indicating potential unidentified cross-reactive components. 

Approximately 20% of experts reported having seen patients with jackfruit as a trigger for allergic reactions, yet diagnostic testing was infrequent. Experts rated the future risk of allergic reactions from jackfruit as the lowest among the surveyed food products, with a slight increase in perceived potential relevance over time. Jackfruit allergy has been linked to Bet v 1-related sensitization, often presenting as pollen-food allergy syndrome, with a 17-kDa PR-10 protein identified as the major allergen [35, 36, 37]. Cases of jackfruit-induced anaphylaxis, particularly in birch pollen-sensitized individuals, have been reported [9, 17]. Anaphylaxis after dried jackfruit consumption has also been observed among latex-allergic individuals [38, 39]. Although clinical data remain limited, the rising consumption of tropical fruits and the growing use of jackfruit as a vegan meat substitute highlight its potential allergenicity, including cross-reactivity patterns, warranting further research. 

The data on exposure to uncommon pollen and venom allergies reflects climate-driven changes. The spread of invasive species such as *Ambrosia artemisiifolia* or *Vespa velutina*, along with extended pollen seasons, has been linked to heightened symptom burden and sensitization in Europe [40]. 

Most experts were from German-speaking countries, which may limit the generalizability of our findings to other regions in Europe with different healthcare systems or dietary habits. Data were collected only in 2023 and 2025, and therefore temporal trends may be incompletely captured. 

In conclusion our data underline that shifts in nutrition, climate, and biodiversity have reached allergy practicing centers. Specialists not only need to be aware of emerging allergens and their clinical implications, but are also in need of standardized testing methods. 

## Authors’ contributions 

LF managed data acquisition, performed the analysis, and drafted the manuscript. VH contributed to study design, data acquisition, data analysis, and interpretation of results. SDB contributed to study design, data acquisition, and interpretation of results. MW conceived and designed the study, supervised the analysis, and assisted in writing the manuscript. All authors critically revised the manuscript and approved the final version. 

## Funding 

The Anaphylaxis Registry is supported by the Network for Online Registration of Anaphylaxis (NORA e.V.). 

## Conflict of interest 

LF and VH declare no conflict of interest. SDB and MW receive funding by the German Federal Ministry of Education and Research (01EA2107B) and by the German Research Association (Deutsche Forschungsgemeinschaft (DFG) as part of the clinical research unit CRU339: Food Allergy and Tolerance FOOD@ (409525714). MW reports honoraria and/or consulting fees from AbbVie, Aimmune Therapeutics, ALK-Abelló, Allergopharma, Almirall, Amgen, Biotest, Boehringer Ingelheim, DBV Technologies, Genzyme, LEO Pharma, Eli Lilly, Viatris, Novartis, Pfizer, Regeneron Pharmaceuticals Inc., Sanofi, Stallergenes Greer. 

**Table 1.** Baseline characteristics of experts.

**Characteristic**	**N = 127**
Profession	
Physician	108 (86%)
Nutritionist	17 (13%)
Psychologist	1 (0.8%)
Years of expertise	16 (5, 28)
Country	
Germany	48 (38%)
Austria	36 (29%)
Spain	8 (6.3%)
Switzerland	7 (5.6%)
United Kingdom	5 (4.0%)
Poland	5 (4.0%)
France	4 (3.2%)
Other	14 (9.9%)
Gender, female	92 (72%)
Patient focus	
Food	112 (88%)
Pollen	75 (59%)
Insect	58 (46%)
Drug	56 (44%)
Age groups of patients	
Children^a^	15 (12%)
Older children^b^	57 (45%)
Adults^c^	32 (25%)
All age groups	28 (22%)

n (%); median (IQR). ^a^≤ 10 years; ^b^11 – 17 years; ^c^≥ 18 years.

**Table 2.** Experts’ counselling on potential cross-reactivity.

	**Cross-reactivity advice**			
	**Edible insects, N = 125**	**Hemp seeds/cannabis, N = 126**	**Jackfruit, N = 125**	Legumes*, N = 126
Response	54 (43%)	33 (26%)	10 (8.0%)	90 (71%)

^1^n (%). *Legumes other than peanut/soy/lupine. Relevant at-risk patient groups: legumes* – peanut/soy/lupine allergy; edible insects – house dust mite/seafood allergy; hemp seeds/cannabis – non-specific lipid transfer protein syndrome; jackfruit – birch pollen allergy.

**Table 3.** Changes in pollen and venom allergen exposure.

**Characteristic**	**N = 127**
Pollen allergies	
Allergies due to novel pollen	34 (34%)^a^
Problems due to longer pollen season	83 (77%)^b^
Insect venom allergies	
Allergies due to novel stinging insects	21 (19%)^c^
Problems due to longer insect season	30 (30%)^d^

n (%); ^a^Data available from n = 100; ^b^data available from n = 108; ^c^data available from n = 110; ^d^data available from n = 99.

## Supplemental material

Supplemental material Questionnaire and Supplemental Figure.
