# Supplementary material for: Experts’ perspectives on allergic reactions to emerging food sources, pollen and insects
Source: Allergol Select. 2026 Mar 17;10:28–35. doi: 10.5414/ALX02611E (PMC13010207; doi:10.5414/ALX02611E)
Supplement: Supplemental material [file allergologieselect-10-028-S01.pdf]

Experts' perspectives on allergic reactions to emerging food sources, pollen and insects

Online Repository:

Questionnaire:

Dear Anaphylaxis-Experts,

Welcome to Berlin! We are happy to meet you in person and we are looking forward to interesting talks, presentations, and discussions.

One of the major topics over the recent years is climate change and how we can minimize the human impact on the environment. One subject under discussion is human nutrition, especially the consumption of meat and other animal products.

Recently, insects have been authorized as novel foods by the European Food Safety Authority and can now be added to different foods like pasta, crisps, or chocolate. In addition, plant-based protein sources like peas, lentils, soy or peanuts are on the rise. Some of them do not legally require labelling, leading to potential problems for patients with allergies to the specific food. Another newly upcoming protein source is hemp, which can cause allergic reactions in patients with LTP sensitization. With this questionnaire, we want to find out, to what extent you see patients with allergic reactions to or questions about novel foods and other aspects of allergies that relate to climate change and changes in our diet.

1) **In which country do you practice?** ______________________________________

2) **For how many years are you practicing in allergology?** ________________________________

3) **Please indicate your gender:** ☐ male ☐ female ☐ intersexual/ diverse

4) **Please indicate your profession:** ☐ physician ☐ nutritionist ☐ patient organization

5) **What is your major patient focus? (Multiple answers possible)**

   Elicitors:
   - ☐ Insect allergy
   - ☐ Drug allergy
   - ☐ Food allergy
   - ☐ Pollen allergy
   - ☐ Other, please specify: __________________

   Age groups:
   - ☐ Children, max. 10 years of age
   - ☐ Children & adolescents
   - ☐ Adults
   - ☐ All age groups

6) **What is your specialty?**
   - ☐ Dermatology
   - ☐ Paediatrics
   - ☐ Pneumology
   - ☐ Allergology (incl. paediatric allergology)
   - ☐ Other, please specify ____________________________

7) If you see food allergic patients:

How many food allergic patients do you approximately see

-per month? ____________ [number]

-per year? ____________ [number]

With the following questions, we want to ask about your experience and expectations about allergies to novel foods or foods that are increasingly used over the recent years:

8) Edible insects (e.g. yellow mealworm, migratory locust, grain mould beetle, domestic cricket)

a) Have you ever seen patients who reported an allergic reaction to edible insects?

- ☐ No, never
- ☐ Yes, once
- ☐ Yes, around 1 patient/year
- ☐ Yes, around 2 - 5 patients/year
- ☐ Yes, more than 5 patients/year

b) Do your patients ask you about any allergy risks regarding edible insects?

- ☐ No, never
- ☐ Yes, once
- ☐ Yes, around 1 patient/year
- ☐ Yes, around 2 - 5 patients/year
- ☐ Yes, more than 5 patients/year

c) Have you ever performed a skin prick test with edible insects?

- ☐ No, never
- ☐ Yes, once
- ☐ Yes, in around 1 patient/year
- ☐ Yes, in around 2 - 5 patients/year
- ☐ Yes, in more than 5 patients/year

If yes: Was the skin prick test with edible insects positive?

- ☐ No/none of them
- ☐ Some of them
- ☐ All of them

d) Do you regularly perform skin prick test for Dermatophagoides pteronyssinus/ farina?

- ☐ Yes, in all patients with indication for a skin prick test, irrespective of the indicating allergen
- ☐ Yes, but only in patients with an indication for skin prick test to house dust mite
- ☐ No, I don’t perform skin prick test with Dermatophagoides pteronyssinus/ farina
- ☐ Other, please specify: ____

e) Have you ever performed food challenges with edible insects?

- ☐ No
- ☐ Yes

If yes: Was the challenge with edible insects positive?

- ☐ No/none of them
- ☐ Some of them
- ☐ All of them

f) Do you advice patients with a known allergy to crustaceans about the potential risk of crossreac-tivity to edible insects?
- ☐ No
- ☐ Yes

g) To what extent do you think allergy/anaphylaxis to edible insects will increase following the legal authorization of insects for use as/in food products?

0 1 2 3 4 5 6 7 8 9 10

No Very high

9) Jackfruit

a) Have you ever seen patients who reported an allergic reaction to jackfruit?
- ☐ No, never
- ☐ Yes, once
- ☐ Yes, around 1 patient/year
- ☐ Yes, around 2 - 5 patients/year
- ☐ Yes, more than 5 patients/year

b) Do your patients ask you about any allergy risk regarding jackfruit?
- ☐ No, never
- ☐ Yes, once
- ☐ Yes, around 1 patient/year
- ☐ Yes, around 2 - 5 patients/year
- ☐ Yes, more than 5 patients/year

c) Have you ever performed a skin prick test with jackfruit?
- ☐ No, never
- ☐ Yes, once
- ☐ Yes, in around 1 patient/year
- ☐ Yes, in around 2 - 5 patients/year
- ☐ Yes, in more than 5 patients/year

If yes: Was the skin prick test with jackfruit positive?

- ☐ No/none of them
- ☐ Some of them
- ☐ All of them

d) Have you ever performed food challenges with jackfruit?
- ☐ No
- ☐ Yes

If yes: Was the challenge with jackfruit positive?

- ☐ No/none of them
- ☐ Some of them
- ☐ All of them

e) Do you advice patients with a known allergy to birch-pollen about the potential risk of an allergic reaction to jackfruit?
- ☐ No
- ☐ Yes

f) To what extent do you think allergy/anaphylaxis to jackfruit will increase following the upcoming use of jackfruit as an alternative to meat?

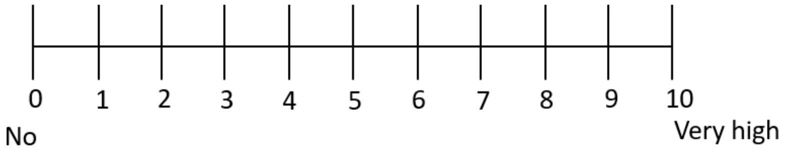

10) Hemp Seeds/Cannabis

a) Have you ever seen patients who reported an allergic reaction to hemp seeds/cannabis?
- ☐ No, never
- ☐ Yes, once
- ☐ Yes, around 1 patient/year
- ☐ Yes, around 2 - 5 patients/year
- ☐ Yes, more than 5 patients/year

b) Do your patients ask you about any allergy risks of hemp seeds/cannabis?
- ☐ No, never
- ☐ Yes, once
- ☐ Yes, around 1 patient/year
- ☐ Yes, around 2 - 5 patients/year
- ☐ Yes, more than 5 patients/year

c) Have you ever performed a skin prick test with hemp seeds/cannabis?
- ☐ No, never
- ☐ Yes, once
- ☐ Yes, in around 1 patient/year
- ☐ Yes, in around 2 - 5 patients/year
- ☐ Yes, in more than 5 patients/year

If yes: Was the skin prick test with hemp seeds/cannabis positive?

- ☐ No/none of them
- ☐ Some of them
- ☐ All of them

d) Have you ever performed food challenges with hemp seeds/cannabis?
- ☐ No
- ☐ Yes

If yes: Was the challenge positive?

- ☐ No/none of them
- ☐ Some of them
- ☐ All of them

e) Do you advice patients with a known LTP-syndrome about the potential risk of an allergic reaction to hemp seeds/cannabis?
- ☐ No
- ☐ Yes

f) To what extent do you think allergy/anaphylaxis to hemp seeds/cannabis will increase following the planned legal authorization of cannabis and the increased use of hemp as a protein source in food products?

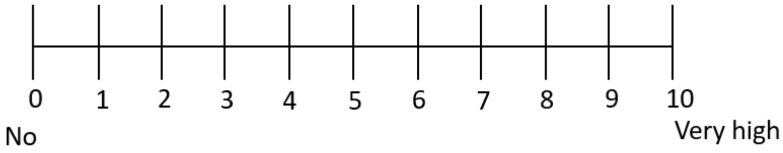

11) Legumes, that do not legally require allergen-specific labelling (e.g. peas, chickpeas, lentils, fenugreek)

Other than peanuts, soy and lupine, they do not belong to the 14 food groups that have to be declared as allergens on menus, in restaurants or highlighted on prepacked foods. Their use as environmentally friendly protein source for meat or dairy product-alternatives increases.

a) Have you ever seen patients who reported an allergic reaction to legumes other than peanut, soy or lupine?
- ☐ No, never
- ☐ Yes, once
- ☐ Yes, around 1 patient/year
- ☐ Yes, around 2 - 5 patients/year
- ☐ Yes, around 5 – 10 patients/year
- ☐ Yes, around 11 – 20 patients/year
- ☐ Yes, more than 20 patients/year

b) Do your patients ask you about any allergy risk regarding legumes other than peanut, soy or lupine?
- ☐ No, never
- ☐ Yes, once
- ☐ Yes, around 1 patient/year
- ☐ Yes, around 2 - 5 patients/year
- ☐ Yes, around 5 – 10 patients/year
- ☐ Yes, around 11 – 20 patients/year
- ☐ Yes, more than 20 patients/year

c) Have you ever performed a skin prick test with legumes other than peanut, soy or lupine?
- ☐ No, never
- ☐ Yes, once
- ☐ Yes, in around 1 patient/year
- ☐ Yes, in around 2 - 5 patients/year
- ☐ Yes, in around 5 – 10 patients/year
- ☐ Yes, in around 11 – 20 patients/year
- ☐ Yes, in more than 20 patients/year

If yes: Was the skin prick test to the specified legumes positive?

- ☐ No/none of them
- ☐ Some of them
- ☐ All of them

d) Have you ever determined specific IgE values for legumes other than peanut, soy or lupine?
- ☐ No, never
- ☐ Yes, once
- ☐ Yes, in around 1 patient/year
- ☐ Yes, in around 2 - 5 patients/year
- ☐ Yes, in around 5 – 10 patients/year
- ☐ Yes, in around 11 – 20 patients/year
- ☐ Yes, in more than 20 patients/year

If yes: Were the specific IgE values for the specified legumes positive?

- ☐ No/none of them
- ☐ Some of them
- ☐ All of them

e) Have you ever performed food challenges to legumes other than peanut, soy or lupine?
- ☐ No
- ☐ Yes

If yes: Was the challenge to the specified legumes positive?

- ☐ No/none of them
- ☐ Some of them
- ☐ All of them

f) Do you advice patients with a known allergy to peanut, soy or lupine about the potential risk of allergy to legumes other than peanut, soy or lupine?

☐ No
☐ Yes

g) To what extent do you think allergy/anaphylaxis to legumes other than peanut, soy or lupine will increase following increased use in food products?

0 1 2 3 4 5 6 7 8 9 10
No Very high

Further research on rare elicitors of anaphylaxis

12) Would you be interested to participate in a new project about novel foods/ rare elicitors via the Anaphylaxis Registry?

☐ No
☐ Yes

Pollen allergies

13) If you see pollen allergic patients:

How many pollen allergic patients do you approximately see

-per month? ____________ [number]

-per year? ____________ [number]

14) Do you see patients with allergies to plant that were previously not growing in your country?

☐ No
☐ Yes

If yes, please specify, which novel plants cause the allergies: ______________________

____

15) Do you notice problems in pollen allergic subjects due to longer pollination seasons?

☐ No
☐ Yes

If yes, please specify, which pollen allergies are the most affected: _________________

____

Insect venom allergies

16) If you see insect venom allergic patients:

How many insect venom allergic patients do you approximately see

-per month? ____________ [number]

-per year? ____________ [number]

17) Do you see allergies due to novel stinging insects in your country?

- ☐ No
- ☐ Yes

If yes, please specify the type of novel insects: ___________________________________

____

18) Do you notice problems due to longer insect seasons due to the climate changes (e.g earlier awakening or later winter sleep/cold induced death of stinging insects)?

- ☐ No
- ☐ Yes

If yes, please specify the type of novel insects: ___________________________________

____

Thank you very much for taking the time to fill out this questionnaire!

Your Anaphylaxis-Team

Figure E1

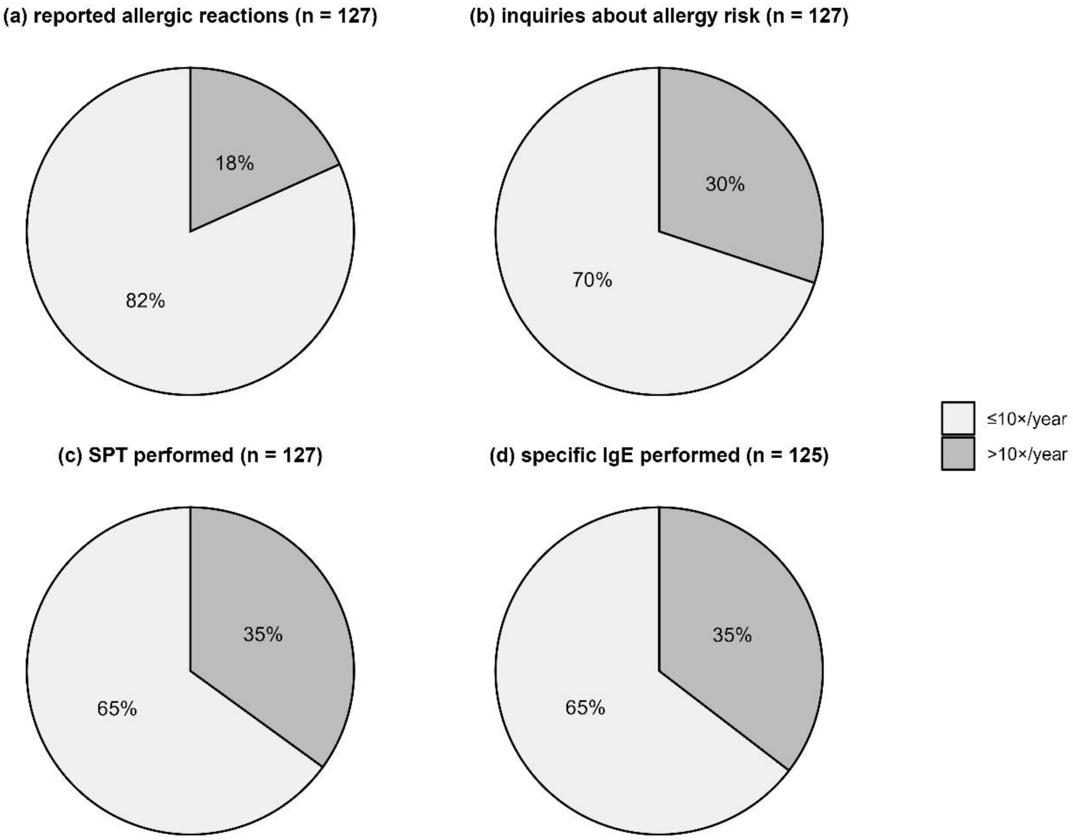

FIGURE E1: Relative frequency of responses regarding legumes*: (a) reported allergic reactions by patients, (b) patients' inquiries about allergic risk, (c) performed skin prick tests (SPT), and (d) performed specific IgE diagnostic. Experts are grouped by those reporting ≤10 versus >10 events per year for each category.
